# A randomized, controlled, crossover pilot study of losartan for pediatric nonalcoholic fatty liver disease

**DOI:** 10.1186/s40814-018-0306-4

**Published:** 2018-06-05

**Authors:** Miriam B. Vos, Ran Jin, Juna V. Konomi, Rebecca Cleeton, Jessica Cruz, Saul Karpen, Dellys Soler Rodriguez, Jennifer K. Frediani, Courtney McCracken, Jean Welsh

**Affiliations:** 1 Division of Gastroenterology, Hepatology, and Nutrition, Department of Pediatrics, School of Medicine, Emory University, Room W-450, 1760 Haygood Dr NE, Atlanta, GA 30322 USA; 2 Children’s Healthcare of Atlanta, Atlanta, GA USA; 3 Department of Pediatrics, School of Medicine, Emory University, Atlanta, GA USA

**Keywords:** Children, Treatment, Pilot, Fatty liver disease, Plasminogen activator inhibitor-1, Insulin resistance

## Abstract

**Background:**

Nonalcoholic fatty liver disease (NAFLD) is the most common liver disease in children, and currently, there are no FDA-approved therapies. Plasminogen activator inhibitor-1 (PAI-1) is elevated in children with NAFLD and associated with increased disease severity. Losartan potassium (losartan) is an angiotensin II receptor blocker (ARB) that reduces PAI-1 production and improves insulin sensitivity that has been proposed as a treatment for pediatric NAFLD but has not previously been tested.

**Methods:**

This was an 8-week randomized, double-blind, placebo-controlled, phase 2a, crossover study (with a 6-week washout between conditions) for safety and preliminary efficacy of losartan 50 mg a day taken orally in 12 normotensive children with biopsy proven nonalcoholic steatohepatitis (NASH).

**Results:**

Twelve children enrolled in the study, and nine completed all visits. No changes in blood pressure or serious adverse events occurred during the study. Trends in improvement in alanine aminotransferase (ALT), aspartate aminotransferase (AST), and homeostatic model assessment insulin resistance (HOMA-IR) were seen with losartan treatment compared to the placebo time-period. More participants decreased ALT on losartan as compared to placebo (89% [8 out 9] vs. 56% [5 out of 9], respectively).

**Conclusions:**

This data provides preliminary evidence that losartan treatment is safe over 8 weeks in children with NAFLD and supports consideration of larger studies to test its efficacy.

**Trial registration:**

URL and trial identification number: https://clinicaltrials.gov/show/NCT01913470, NCT01913470.

Date registered: August 1, 2013.

## Background

Nonalcoholic fatty liver disease (NAFLD) is the leading cause of liver disease in children [1]. The clinical importance of NAFLD extends beyond liver injury to include increased cardiovascular disease [2], type 2 diabetes [3], and increased overall mortality [4]. Our work and others have demonstrated that adolescents with NAFLD have increased traditional markers of cardiovascular disease (CVD) such as increased triglycerides, low high-density lipoprotein (HDL), and post-prandial lipemia [5, 6]. Current treatment recommendations include lifestyle changes, including attempting to improve diet and increase physical activity; however, lifestyle changes typically do not achieve resolution of NAFLD [7].

Plasminogen activator inhibitor-1 (PAI-1) is an acute-phase protein which increases in states of insulin resistance, inflammation, and injury [8] and is elevated in both adults and children with hepatic steatosis [9–11]. Furthermore, PAI-1 correlates with fibrosis stage [12, 13]. The renin-angiotensin system (RAS) has been suggested to be involved in liver damage pathways and might play a critical role in the pathogenesis of NAFLD [14]. Blockage of the RAS significantly inhibits the expression of PAI-1 in the liver [15], and angiotensin receptor blockers (ARB), a class of medications that antagonize the angiotensin receptor and suppress RAS, have been proposed as a novel treatment for NAFLD in part not only because they decrease PAI-1 but also because they improve insulin resistance [16]. No data is available on RAS blocking medications in children with NAFLD, although losartan (an ARB) is approved for treatment of hypertension in children.

Because no data existed for losartan in children with NAFLD, a phase 2a, proof of concept study was designed. We completed an 8-week, randomized, double-blind, placebo-controlled, pilot crossover trial aiming to test preliminary efficacy and safety of losartan potassium (losartan, an ARB) on pediatric normotensive NAFLD patients.

## Methods

This was a randomized, double-blind, placebo-controlled, crossover study (phase IIa clinical therapeutic development trial) that aimed to establish the safety and efficacy of losartan in a new NAFLD population. This study was approved by the Emory University Institutional Review Board and listed on Clinical Trials.gov (NCT01913470). Adolescents with biopsy proven NASH, who failed to normalize liver enzymes with conventional therapy (attempted lifestyle changes including healthier diet and physical activity), were consented and assessed for enrollment eligibility at a screening visit. The liver biopsies were obtained during routine clinical care and assessed by one of several hospital pathologists. The inclusion criteria were age 11–19 years at enrollment; body weight ≥ 62.5 kg; BMI > 85th for age and gender; history of definite or borderline NASH based upon histology using NASH clinical research network (CRN) criteria [17]; ALT ≥ 3 times normal (69 U/L for girls, 78 U/L for boys) at enrollment; and at least 2 months of attempted lifestyle changes after liver biopsy. Exclusion criteria were history of cirrhosis and liver synthetic dysfunction (international normalized ratio (INR) ≥ 1.5); history of hypotension; diagnosis of diabetes (or fasting glucose > 125 mg/dl); renal insufficiency (glomerular filtration rate (GFR) < 30); any other chronic disease requiring daily medication (except medications for acid reflux, allergies or asthma); acute illness within past 2 weeks prior to enrollment (fever > 100.4 °F); and any anti-oxidant therapy or supplements within past 4 weeks before enrollment.

After a 4-week stabilization period, subjects were randomized to losartan or placebo for 8 weeks followed by a 6-week washout and then the alternate therapy for 8 weeks using a randomized code prepared by the statistician and placed in sealed envelopes. Participants, investigators, and coordinators were blinded to the intervention throughout the study, and the pills appeared identical. Participants’ study visits were at weeks 4, 8, 12, 14, 22, and 28 for safety monitoring as well as intermediate efficacy. Fasting blood samples were collected (typically between 7 and 9 am) at weeks 0, 8, 14, and 22. All participants continued their usual healthy diet and exercise as recommended by their NAFLD physician and were asked not to make major changes during the study. Patients started losartan or identical placebo pills at 25 mg per day for 1 week and 50 mg for 7 weeks, and then the alternate therapy for 8 weeks (25 mg daily for 1 week and 50 mg daily for 7 weeks) after a 6-week washout. The primary side effect associated with losartan is reduction of blood pressure. To ensure safety, parents were provided and taught to use an automated blood pressure cuff for home monitoring of blood pressure.

### Laboratory measurements

Concentrations of ALT and AST were measured at Children’s Healthcare of Atlanta clinical laboratory. Freshly collected plasma samples were protected from light and transported on ice pack for further processing using AU480 chemistry analyzer (Beckman Coulter, Inc.). Plasma glucose levels were measured by enzymatic methods (Beckman Diagnostics, Fullerton, CA) and insulin was determined using immunoturbidometric methods (Sekisui Diagnostics, Exton, PA). HOMA-IR, the homeostatic model assessment for insulin resistance index, was calculated as fasting glucose (mg/dl) × insulin (mU/L)/405. Plasma PAI-1 concentrations were measured in duplicate using a commercially available enzyme-linked immunosorbent assay kit (Catalog# KHC3071) from Thermo Fisher Scientific (Waltham, MA), according to the manufacturer’s instruction.

### Statistical analysis

Because this was a feasibility study, the sample size was modeled after other pilot pediatric NAFLD studies [18, 19]. A well-powered trial of ALT in pediatric NAFLD typically requires > 70 patients per arm, and given the lack of preliminary and safety data for losartan in children with NAFLD, we selected to include 12 subjects and to examine safety, tolerability and preliminary efficacy. The primary outcome was change in ALT level.

After the study was completed, we tested for carryover and crossover effects for both outcomes ALT and AST. Significance of the carryover effect was tested at the 0.1 level, and analyses were conducted using SAS v.9.4 (Cary, NC). Critically, an unexpected, significant carryover effect of losartan was detected in ALT, the primary outcome, meaning that participants did not return their ALT concentrations to baseline levels. Because of the carryover of improvement into the second treatment period, it was determined to be statistically incorrect to compare combined treatment groups as previously planned in the study design. Raw data at the individual patient level is provided.

## Results

Twelve children were enrolled in the study; however, data from ten participants was available for the analysis. Three participants withdrew from the study due to noncompliance (*n* = 1), self-withdrawal (*n* = 1), and loss to follow-up (*n* = 1). Nine subjects completed all time points (Table 1). Enrollment continued from 2013 to 2015. Data from these participants was used if “pre” and “post” values of a period treatment were available. Sixty-seven percent of subjects were Hispanic with mean age of 14 years and BMI *z*-score of 2.32. No significant weight or BMI change was observed in the losartan vs. placebo time periods during the study (*p* > 0.05). There were no serious adverse events reported and adverse events were balanced between treatment periods.

**Table 1:** Descriptive variables (*N* = 9)

Parameters	Mean (SD)
Age (years)	13.8 (1.99)
Male (*n*, %)	5 (55.6)
Hispanics (*n*, %)	7 (77.8)
BMI *z*-score	2.25 (0.43)
SBP (mm Hg)	121 (10.7)
DBP (mm Hg)	73.9 (5.90)
ALT (U/L)^a^	90.0 (61.0)
AST (U/L)	55.3 (12.2)
GGT (U/L)	37.7 (16.0)
HOMA-IR^a^	10.1 (23.5)
Triglyceride (mg/dl)	83.0 (28.5)
HDL (mg/dl)	44.5 (4.39)
PAI-1 (ng/ml)	5.56 (2.01)
NASH (%)	6 (66.7%)

^a^Values are median (IQR)

**Fig. 1:**
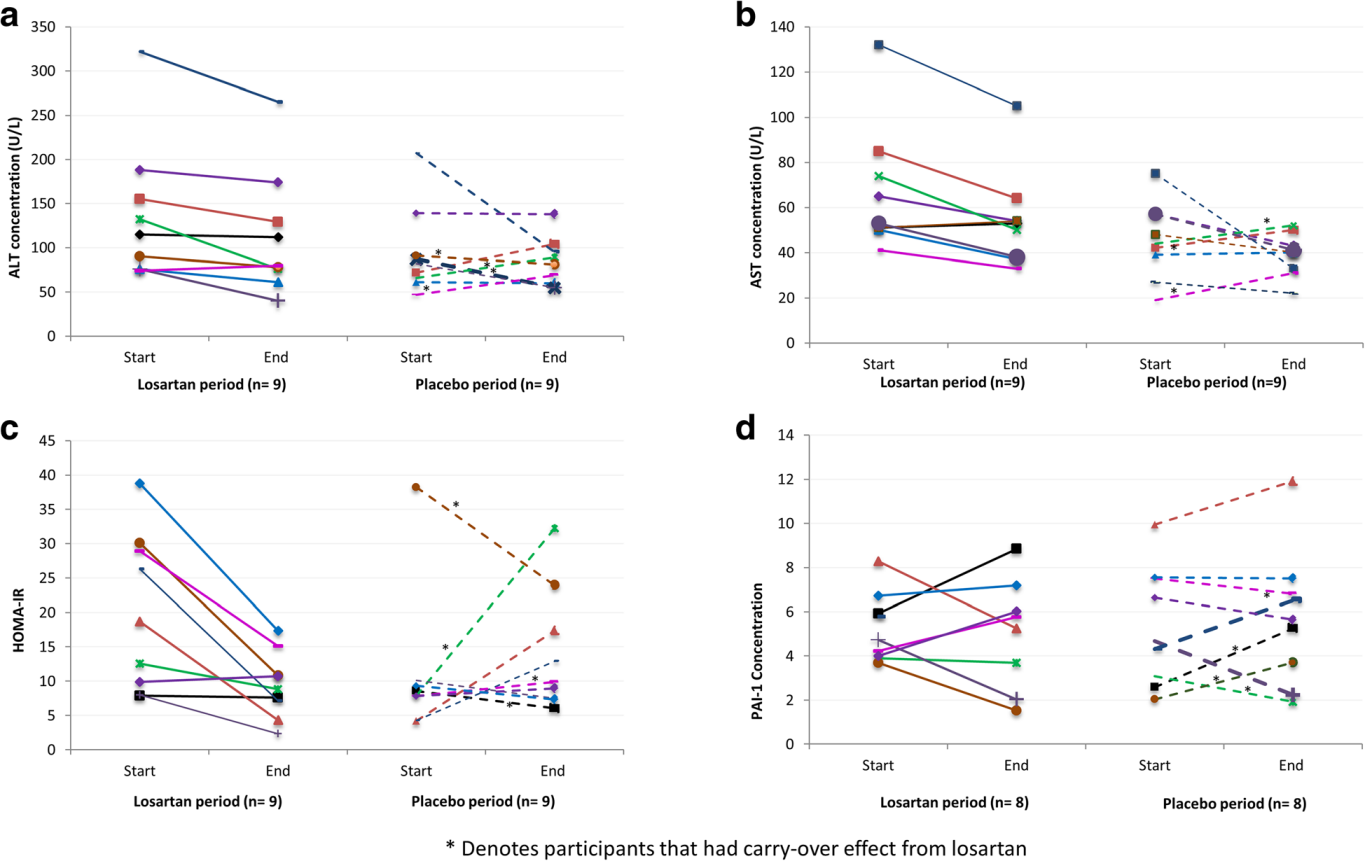
**a**–**d** Concentrations of liver enzymes, PAI-1 and HOMA-IR pre- and post-treatment periods

Carryover effect analysis showed that participants did not return to baseline after the first 8-week period of treatment for the primary measured outcome, ALT concentration. Participants that were initially randomized to losartan continued to decline even after they crossed over to the placebo treatment, while the majority of participants initially randomized to placebo had higher ALT at the start of the second treatment (week 14, losartan) than at baseline. No changes in blood pressure were observed within and between losartan and placebo periods for the duration of the study. The systolic blood pressures were 122.8 ± 11.6 mmHg and 117.0 ± 16.7 mmHg pre- and post-losartan period and 120.1 ± 8.7 mmHg and 121.6 ± 5.4 mmHg pre- and post-placebo period, respectively. The diastolic blood pressures were 71.6 ± 7.8 mmHg and 71.4 ± 10.4 mmHg, pre- and post-losartan period, and 70.3 ± 6.4 and 71.1 ± 6.1 pre- and post-placebo period. Individual changes in ALT, AST and HOMA-IR and PAI-1 over 8 weeks of losartan and placebo periods are shown in Fig. 1a–d. More participants decreased ALT while on losartan as compared to while on placebo (89% [8 out 9] vs. 56% [5 out of 9], respectively). For AST, 78% of participants (7 out of 9) had decreased AST concentrations by week 8 while on losartan, while in the placebo period 44% (4 out of 9) had decreased AST by week 8 of treatment. At week 8 of the losartan period, most participants showed improvement in insulin resistance, as measured by HOMA-IR (78% [7 out of 9]) while after the placebo period this number dropped to 44% (4 out of 9). No difference was seen in PAI-1 from baseline to end of each treatment (Fig. 1d).

## Discussion

This is a brief report of an early phase clinical trial testing preliminary efficacy and safety and evaluating other biologic response variables. Losartan was found to be well tolerated by pediatric NAFLD participants, despite their normotensive state at baseline. We saw a promising trend in improvement of both insulin resistance and liver enzymes. Interestingly, there was no change in PAI-1 concentrations, despite this being an expected effect of losartan. Our findings of improved insulin resistance in this pediatric cohort are in accordance with previous studies in adults in which treatment with losartan reduces insulin resistance.[20] Losartan treatment is thought to improve insulin resistance through direct effects on the RAS system [20] rather than suppression of PAI-1, which is consistent with our results.

This was a phase 2a pilot study and therefore was intentionally small and short. We utilized a crossover design to minimize inter-subject variability and increase the study power. However, there was an unexpected carryover effect of losartan in those randomized to losartan first, despite the 6-week washout period. Previous pediatric pharmacokinetic studies of losartan did not show carryover effect past 2 weeks [21]. The carryover in ALT improvement from losartan over 6 weeks after going off treatment was unfortunate for the analysis of this phase 2a study, but it demonstrates strong justification for further evaluation of losartan, as sustained improvement in ALT and insulin resistance from a medication is a benefit to children with NAFLD. A major strength of this study is that extensive monitoring of blood potassium levels and blood pressures were carried out throughout the study with no adverse events observed. Larger, later phase studies will be needed to determine longer-term safety and efficacy of losartan.

## Conclusions

In summary, losartan treatment for 8 weeks was safe in a pediatric normotensive NAFLD cohort, with no serious adverse events reported and stable blood pressure throughout the study. We found preliminary trends of improved ALT and insulin resistance by losartan supporting further testing of losartan for pediatric NAFLD in a larger, randomized controlled trial with parallel groups.

## Abbreviations

ALT: Alanine aminotransferase
ARB: Angiotensin receptor blockers
AST: Aspartate aminotransferase
CRN: Clinical research network
CVD: Cardiovascular disease
GFR: Glomerular filtration rate
HDL: High-density lipoprotein
HOMA-IR: Homeostatic model assessment insulin resistance
INR: International normalized ratio
NAFLD: Nonalcoholic fatty liver disease
NASH: Nonalcoholic steatohepatitis
PAI-1: Plasminogen activator inhibitor-1
RAS: Renin-angiotensin system
